# Assessing the uptake of persistent identifiers by research infrastructure users

**DOI:** 10.1371/journal.pone.0175418

**Published:** 2017-04-10

**Authors:** Matthew S. Mayernik, Keith E. Maull

**Affiliations:** NCAR/UCAR Library, National Center for Atmospheric Research (NCAR), University Corporation for Atmospheric Research (UCAR), Boulder, Colorado, United States of America; University of Helsinki, FINLAND

## Abstract

Significant progress has been made in the past few years in the development of recommendations, policies, and procedures for creating and promoting citations to data sets, software, and other research infrastructures like computing facilities. Open questions remain, however, about the extent to which referencing practices of authors of scholarly publications are changing in ways desired by these initiatives. This paper uses four focused case studies to evaluate whether research infrastructures are being increasingly identified and referenced in the research literature via persistent citable identifiers. The findings of the case studies show that references to such resources are increasing, but that the patterns of these increases are variable. In addition, the study suggests that citation practices for data sets may change more slowly than citation practices for software and research facilities, due to the inertia of existing practices for referencing the use of data. Similarly, existing practices for acknowledging computing support may slow the adoption of formal citations for computing resources.

## Introduction

Research organizations, data repositories, and universities are now assigning persistent web-accessible identifiers in large numbers to scientific resources such as data sets, software packages, and research facilities. Most such efforts are utilizing identifier assignment services provided by the DataCite organization (http://datacite.org; see also [1,2]), which enables individual organizations to register Digital Object Identifiers (DOI) for digital and non-digital resources [3,4]. Assigning these persistent identifiers serves two crucial purposes. First, persistent identifiers increase the traceability and reusability of scientific resources by providing a means to persistently link to data sets, software, and other resources within the scientific literature. Second, without such traceability, it is difficult, if not impossible to measure the impact such resources have within the communities they belong to, or to understand the spread of that impact to broader scientific communities.

Significant progress has been made in the past few years in the development of recommendations, policies, and procedures for creating and promoting persistent identifiers, ranging from general recommendations [5], to recommendations from discipline-specific groups [6,7]. In addition, multiple international interest groups came together to create a consensus set of data citation principles [8]. These principles were made public in February of 2014, and, as of September 2016, have been endorsed by more than 110 organizations.

Open questions remain, however, about the success of these initiatives. Are the referencing practices of authors of scholarly publications changing to incorporate these newly assigned identifiers to research infrastructural resources? This paper uses four focused case studies to evaluate this issue, namely, whether research resources are being increasingly identified and referenced in the research literature via persistent web-accessible identifiers. We provide empirical evidence to help answer a set of key questions:

- Do paper authors reference resources more consistently when persistent IDs have been assigned?
- How are citations and/or acknowledgments to scientific resources changing over time in relation to the assignment of IDs?

This paper provides an assessment of the initial uptake of data, software, and facility citation within a particular scientific community. Our findings should inform continued discussion about the aforementioned data citation principles and tools, in particular, about how those principles and tools manifest in practice.

## Background: From data citation to research infrastructure citation

Although the idea of formal citations to data has been discussed in some communities for at least 30 years [9,10], the majority of the work on data citations has been concentrated in the past decade. Data citation promotes a simple concept: researchers who use data to produce a scholarly publication should provide references to the data that they used, just like they provide references to traditional scholarly resources—books, journal articles, reports, and other texts. Persistent identifiers like DOIs have been central to the data citation concept from the start of the recent wave of such initiatives [11]. Kunze [12] refers to DOIs and other similar identifier systems as “actionable” identifiers, because they provide a web-based mechanism to locate the resource being identified. This location feature is what differentiates DOIs and other such identifier systems from identifiers that are used for internal asset tracking within databases or file management systems [13].

Data citation, however, has proven to be much more complex to implement than appears at first glance. Questions quickly arise about data set identity, the inertia of current research practices, and the sustainability of the data being cited [14]. Each data citation initiative needs to make decisions about what is being identified, who should be attributed as authors or contributors, when identifiers should be assigned, and how resources and their associated identifiers will be managed and maintained over time [10]. These challenges have stimulated much discussion. Implementation recommendations provide guidance on how to make progress, even if many issues are very context-dependent [15,16].

As data citation initiatives have gained more prominence, interest has grown for similar initiatives for other scholarly resources, including for software, geological physical samples, biological laboratory resources, and research facilities [17,18,19]. We use the term “research infrastructures” as an overarching grouping of multiple kinds of resources, building on terminology established in international contexts [20,21]. “Research infrastructures” include scientific equipment, collections and archives of scientific data or software, computing systems, and any other tools that enable research.

Arguments for data citations hold true for many other kinds of research infrastructural components. Citations to software would help make software more findable and accessible for re-use. Similarly, managers of many kinds of research facilities are interested in having better measurements of facility use through counting citations, to demonstrate value of their services and to help improve those services. Assigning persistent web-accessible identifiers to software, laboratory materials, and research facilities has the potential to enable a clearer understanding of how those resources are used, just as data citations have the potential to connect scholarship to underlying data via more robust attribution and acknowledgment of data sources.

## Organizational context

This study is investigating references to resources provided and managed by the University Corporation for Atmospheric Research (UCAR) / National Center for Atmospheric Research (NCAR). NCAR is a Federally Funded Research and Development Center (FFRDC) supported by the US National Science Foundation, based in Boulder, CO. UCAR is a non-profit organization that manages NCAR and provides additional capabilities for the atmospheric and related Earth system science communities. For simplicity, this paper refers to both together as UCAR. A data citation initiative began within UCAR in late 2010, driven by a number of UCAR data management teams who were interested in assigning DOIs to their data sets. An ad hoc committee was started in the summer of 2011. This committee met monthly for about a year, and has met approximately every 2–4 months from 2012 up to the present to develop coherent data citation approaches across the organization. The committee formalized internal recommendations for technical tools and methods, policy/procedural protocols and standards, and user and community engagement [22]. This effort has included broad participation from a number of distinct data management groups within UCAR, and has engaged with external organizations and initiatives as much as possible, leveraging community activity to develop robust internal recommendations.

From the early stages of this ad hoc committee’s activity, interest was expressed by some participants in assigning DOIs or other persistent identifiers to additional kinds of resources, such as software. The first two persistent IDs assigned by UCAR (using the EZID service provided by the California Digital Library, http://ezid.cdlib.org/) were DOIs for the NCAR Command Language (NCL) software package and the North American Regional Climate Change Assessment Program (NARCCAP) dataset. The third ID assigned was an Archival Resource Key (ARK, https://wiki.ucop.edu/display/Curation/ARK; [12]) to the Yellowstone IBM iDataPlex System, a supercomputing resource managed by UCAR. The reason for assigning an ARK to the Yellowstone supercomputer instead of a DOI was that the idea of “citing a supercomputer” was very new, and it was unclear if it would gain much traction with the facility’s user community. ARKs can be deleted, unlike DOIs, so the expectation was that the Yellowstone ARK could be deleted if no references to the Yellowstone facility accrued. As illustrated below, however, Yellowstone users are indeed regularly referencing the facility via the ARK.

Since the assignment of those first three identifiers, UCAR groups have assigned IDs to a wide range of resources. Table 1 shows a breakdown of persistent IDs assigned to different kinds of resources by UCAR groups. The “ResourceType” categories correspond to the controlled list of types allowed by the “ResourceTypeGeneral” attribute in the DataCite metadata schema version 3.1 [23].

**Table 1:** Breakdown of persistent IDs assigned to different kinds of resources by UCAR groups, as of Nov. 30, 2016.

ResourceType	Number of IDs assigned
Dataset	3393
Text	626
PhysicalObject	22
Software	8
Collection	3
Model	2
InteractiveResource	1
Event	1
Service	1
[Resource type not supplied]	1
TOTAL	4058

## Method

Tracking citations or references to research infrastructures is currently very difficult, and is typically done via focused studies that look at a small number of resources [20]. This study uses four case studies to examine citation & reference patterns to different kinds of resources.

- Data–North American Regional Climate Change Assessment Program (NARCCAP) data set, DOI assigned May 3, 2012
- Data–NCEP FNL Operational Model Global Tropospheric Analyses data set, DOI assigned January 30, 2014
- Software–NCAR Command Language (NCL), DOI assigned April 10, 2012
- Facility–Yellowstone supercomputer, ARK assigned May 21, 2012

These four resources represent a convenience sample. These resources are used for this study because they cover three different resource types, and were assigned persistent IDs long enough ago (middle of 2012) for a publication record to have built up. As noted above, the NARCCAP data set, NCL software package, and Yellowstone supercomputer were the first three UCAR-managed resources to be assigned persistent IDs via EZID and DataCite. The majority of UCAR’s DOIs (Table 1) have been registered more recently, and as such have not had time for any publication record to emerge. The NARCCAP data set and NCL software package are also the most accessed DOIs for UCAR resources of their type according to the DOI resolution stats provided by DataCite (http://stats.datacite.org). The fourth case study, the NCEP FNL Operational Model Global Tropospheric Analyses data set (abbreviated to “NCEP FNL data set” for the rest of this paper), is included as an additional comparison case because it has the second highest DataCite DOI resolution stats for a UCAR data set, with approximately a factor of four more resolutions than any other data set during 2014–2015.

These four resources are managed by four different groups in NCAR’s Computational and Information Systems Laboratory (CISL). In addition to assigning these persistent IDs, the managers of each resource posted a recommended citation on their respective web sites. The recommended citations for the NARCCAP and NCEP FNL data sets were based on the ESIP data citation recommendation (Federation of Earth Science Information Partners, 2012). The recommended citations for the NCL software package and the Yellowstone supercomputer were not based on any specific examples, but were constructed to look similar to typical citations. The web site for the Yellowstone supercomputer also lists a possible acknowledgment using the ARK, because the facility managers wanted users to have multiple options for referencing the facility in case authors did not want to include a formal citation, or journals did not allow it. The following list shows the citations posted on the resources’ web sites (as of November 2016).

- NARCCAP Data:
  ○ Mearns, L.O., et al., 2007, updated 2014. The North American Regional Climate Change Assessment Program dataset, National Center for Atmospheric Research Earth System Grid data portal, Boulder, CO. Data downloaded yyyy-dd-mm. [doi:10.5065/D6RN35ST]
- NCL Software:
  ○ The NCAR Command Language (Version 6.3.0) [Software]. (2016). Boulder, Colorado: UCAR/NCAR/CISL/TDD. https://doi.org/10.5065/D6WD3XH5
- Yellowstone Supercomputer [Note: this is one of several citation variations provided, with each variation corresponding to different supercomputer usage allocations.]:
  ○ Computational and Information Systems Laboratory. 2012. Yellowstone: IBM iDataPlex System (Climate Simulation Laboratory). Boulder, CO: National Center for Atmospheric Research. http://n2t.net/ark:/85065/d7wd3xhc
- NCEP FNL Data:
  ○ National Centers for Environmental Prediction/National Weather Service/NOAA/U.S. Department of Commerce. 2000, updated daily. NCEP FNL Operational Model Global Tropospheric Analyses, continuing from July 1999. Research Data Archive at the National Center for Atmospheric Research, Computational and Information Systems Laboratory. https://doi.org/10.5065/D6M043C6. Accessed dd mm yyyy.

Literature searches were conducted using the Google Scholar search index between March-May 2016 to find papers that used these four resources. The search terms shown in Table 2 were used to find papers that explicitly noted their use of the resource. Phrases listed in quotes in Table 2 were searched in Google Scholar as phrases. Because this study looked for explicit evidence that a paper used a given resource, it is certain that the numbers reported in the results section below are undercounts of the actual numbers of papers that have made use of these resources. Any papers that used these resources but did not explicitly note their use were not found via this method. In addition, authors may have noted their use of a resource using different terms or phrases than the ones shown in Table 2. As such, the findings reported below should be considered to be lower bounds of the actual numbers of papers that have made use of each resource.

**Table 2:** Google Scholar search terms and phrases used to find papers that used these resources.

NARCCAP data set	NCL software	Yellowstone supercomputer	NCEP FNL data set
10.5065/D6RN35ST	10.5065/D6WD3XH5	85065/d7wd3xhc	10.5065/D6M043C6
"North American Regional Climate Change Assessment Program data"	ncl.ucar.edu	yellowstone supercomputer	“NCEP FNL Operational Model Global Tropospheric Analyses, continuing from July 1999”
"NARCCAP data"	“NCAR Command Language”	yellowstone super computer	“NCEP FNL Operational Model Global Tropospheric Analyses”
"data from NARCCAP"		yellowstone cisl	rda.ucar.edu/datasets/ds083.2 [the data set’s URL after April 2012]
"data from North American Regional Climate Change Assessment Program"		yellowstone "national center for atmospheric research“	dss.ucar.edu/datasets/ds083.2 [the data set’s URL up to April 2012]
"The North American Regional Climate Change Assessment Program Dataset"		yellowstone "computational and information systems laboratory“	ds083.2
"NARCCAP dataset"		yellowstone ncar	ds083.0 [a file format variant of the NCEP FNL data]
narccap.ucar.edu/data			
[searches were also run that used “model” and “model output” in above queries instead of “data” or “dataset”]			

Each document returned by Google Scholar for these search terms was then examined to identify whether the paper actually used the resource of interest, and if so, where in the paper the use of the resource was noted. Any duplicate documents returned by more than one of the above searches were removed to prevent double counting. This study did not count references that occurred in conference abstracts, posters, or presentations.

Each relevant document was coded with regard to whether the following reference types were present:

1. Citation–inclusion of the resource in the reference list
2. Acknowledgment–mention of the resource in an acknowledgment section
3. In-text reference–mention of the use of the resource in the body text

It was quite common for individual documents to include one, two, or all three of these reference types, in every possible combination. To reduce the complexity of coding for this variability in reference type combinations, the reference types were coded in priority order: 1) citations, 2) acknowledgments, and 3) In-text references. For example, in examining a relevant document for the NARCCAP data set, if a formal citation to the data set was present in the reference list, the document was coded as “citation”, whether or not the data set was also mentioned in the acknowledgments or body of the text. If a document included an acknowledgment to the NARCCAP data set but no formal citation, the document was coded as “acknowledgment,” whether or not the data set was also mentioned in the body of the text. Finally, if the usage of the NARCCAP data set was noted in the body of the text, with no formal citation or acknowledgment given, the document was coded as “in-text reference.” This priority order was used because the use of DOIs for data and other resources is primary intended to support the creation of citations, thus our main interest in citations. Services such as the Thomson-Reuters Data Citation Index are being built on top of data citations. The prioritization of acknowledgments vs. the in-text references involved a few factors. In some similar studies, acknowledgments are considered to be part of the article full text, such as in the data citation analysis of Mayo, Vision, and Hull [24], but for this study acknowledgments were given the second priority because they have been examined as an interesting aspect of scholarly communication themselves in numerous studies (e.g. [25,26]), and some work has been done to develop automated acknowledgment analysis tools [27,28]. In-text references were thus assigned the lowest priority.

Each reference was also coded for the presence or absence of the persistent ID. Finally, the relevant documents were then categorized into primary literature vs. grey literature, with the distinction as follows:

1. Primary literature–articles with DOIs, e.g. journal articles, some conference proceedings, some book chapters
2. Grey literature–documents without DOIs, e.g. conference proceedings, theses, pre-prints, etc.

The “primary” vs. “grey” distinction used here is not meant to be definitive, or in any way declare that the assigning of a DOI assigns a higher degree of importance or value to the associated document. This distinction is purely a functional categorization that relates to the production of the time-series figures shown in the results section below. The dates of publication for the relevant documents used to produce the timeline figures below were gathered by querying the DOI resolution server via a Python script to pull down the metadata associated with each document’s DOI. Thus, any document without a DOI was not included in the timeline figures.

## Results

The results are presented in three ways. First, the overall numbers of references are shown to characterize the relative reference counts for the four case studies. Second, the results are broken down to show the patterns of references by reference type. Third, timeline plots are provided to show how the reference trends have varied over time.

The overall results of this investigation are shown in Table 3. The largest number of relevant documents was found for the NCEP FNL data set, and the smallest number found for the NARCCAP data set. As Table 3 shows, documents that reference the Yellowstone Supercomputer showed a higher rate of references that used the associated persistent ID, with 61% of the relevant documents including the Yellowstone ARK within their paper.

**Table 3:** Overall numbers of relevant documents found.

	References that use the ID		References that do not use the ID		Total docs found	% of Total that use the ID
	*Primary Lit*	*Grey Lit*	*Primary Lit*	*Grey Lit*		
**NARCCAP data**	29	12	144	62	247	17%
**NCEP FNL data**	45	5	543	169	762	7%
**NCL software**	140	27	229	192	588	28%
**Yellowstone SC**	191	27	120	19	357	61%

The two non-data cases, Yellowstone and NCL, both showed a higher percentage than the two data cases of references that included the persistent IDs. The proportions of references found within primary and grey literature are fairly consistent across the cases, between 10 and 30% in all cases except for the documents that reference NCL without using the ID, where the ratio of primary to grey literature is closer to parity.

**Fig 1:**
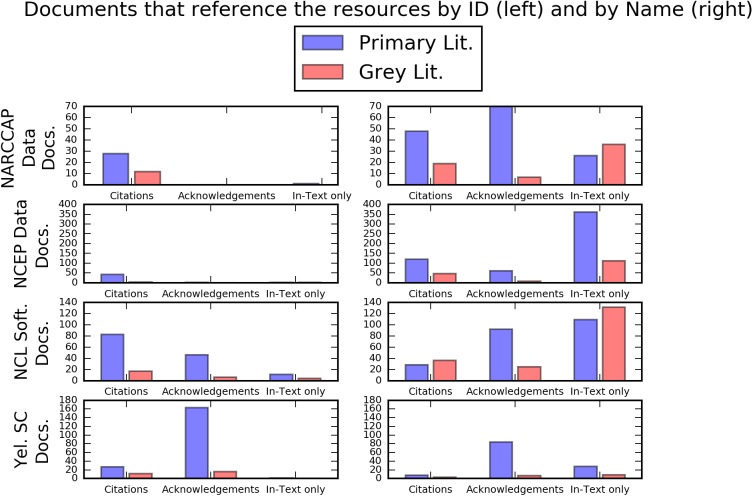
Reference distributions across primary and grey literature. Distribution of Citations, acknowledgments, and In-Text references for the four case studies. Blue bars show references from primary literature, and pink bars show references from grey literature.

Fig 1 shows the breakdown of these overall numbers by the reference type: citation, acknowledgment, and in-text reference. These charts start to show more distinction between the cases, again with the two data cases showing different reference patterns than the two non-data cases. Reference to the NARCCAP and NCEP FNL data sets via their DOIs are almost uniformly citations, whereas references to these data sets via other means, such as their titles or web URLs, show a mix of citations, acknowledgments, and in-text references. The NCEP FNL data set, in particular, shows a very high number of in-text references.

The NCL software reference patterns show a larger proportion of citations for references that use the software package’s DOI. The non-DOI references are predominantly in-text references and acknowledgments. The Yellowstone supercomputer, on the other hand, shows almost the same distribution in both charts, with acknowledgments being by far the most common reference type for references with and without the facilities’ ARK.

**Fig 2:**
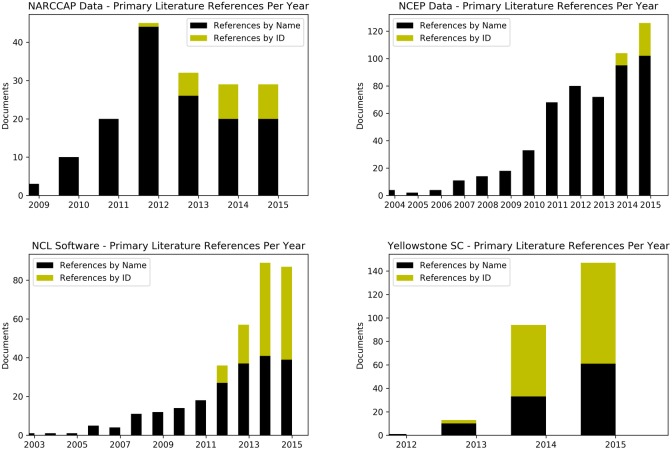
Summation of references over time. Timelines showing the sum of citations, acknowledgments, and in-text references per year from primary literature, with and without the persistent IDs.

**Fig 3:**
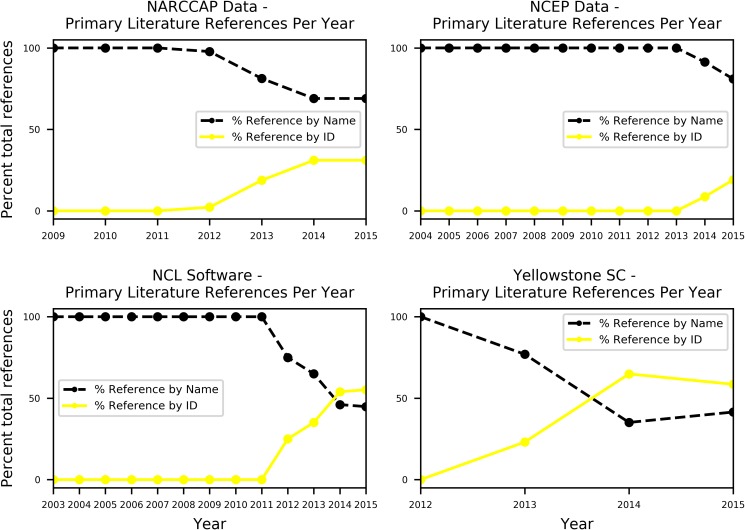
Proportion of references over time. Timelines showing the proportion of primary literature references, with and without the persistent IDs.

Figs 2 and 3 show temporal trends for references to these four case studies with and without their IDs. Fig 2 shows the absolute number of references, and Fig 3 shows the proportion of references to these resources over time. For simplicity, all reference types are lumped together in the timelines to show an overall trend of references with and without the IDs. As noted above, the NCEP FNL DOI was assigned in January 2014. In the other three cases, the ID was assigned in April or May 2012. Fig 2 shows how the absolute numbers of citations have generally increased over time, although a flattening can be seen in the Yellowstone Supercomputer case, and a flattening after a peak in 2012 in the NARCCAP data set case. As shown in Fig 3, the proportion of references that used the persistent IDs have, as a general trend, increased for all resources since the ID was assigned, though again the NARCCAP data set and Yellowstone Supercomputer cases show flattening. These flattening are discussed more below.

For the two non-data examples, references via the persistent identifiers now outnumber references without the identifiers, whereas non-identifier references continue to be prevalent for the data examples. In the case of the NCEP FNL data set, the proportion of references without the DOI has been much larger than the proportion of references that use the DOI. With only two years having passed since the DOI was assigned, however, there is not much trend to assess yet. Based on the other three cases, we would expect a continuous increase in the proportion of references with the DOI vs. without.

The last thing to note about Fig 2 is that there is no clear trend to show that the assignment of a persistent ID has directly led to reductions in the absolute number of references that do not use the persistent ID. Although the NARCCAP data set and NCL software cases suggest such a trend, in the other two cases (NCEP FNL and Yellowstone Supercomputer), the numbers of references that do not use the persistent ID are still increasing at a consistent rate, even if the proportion has decreased as shown in Fig 3.

## Discussion

The results of this study show that the persistent identifiers assigned to the four examined resources are being used in references in published papers on an increasing basis. The identifiers are included in formal references to a large degree for the two data sets examined, but are spread much more widely throughout articles for the two non-data set resources examined, echoing similar findings from a number of prior studies [20]. The shifts in reference practices over time also vary considerably from resource to resource.

A key takeaway from these results is that changing established practices for acknowledging data sets will potentially be more difficult than creating new practices for referencing other kinds of products like software or facilities. For both of the two data sets examined here, less than 40% of the references found included the DOI, even when looking at the most recent year included in the study. This indicates that researchers, when they directly reference the data sets that they have used, are referencing the data sets most commonly by name, web site, or some other means. This relative slow uptake of data citations aligns with findings of researchers who have studied the Thomson-Reuters Data Citation Index, where the majority of the indexed data sets show zero or very few citations [29,30]. This finding, while important, is limited in that it only looks at direct citations, acknowledgments, or in-text references to the data set itself.

An additional factor not directly assessed in this study is that it is common practice for researchers to acknowledge data use by referencing a published paper about the data set (Mayernik, et al., 2015). The NARCCAP data set, in particular, is likely referenced much more frequently via references to published papers than via references directly to the data set. For example, two papers by NARCCAP project investigators each have received many citations (numbers gathered March 3, 2017): Mearns et al. [31] shows 405 citations in the Google Scholar index (Web of Science—N/A), and Mearns et al [32] shows 243 citations in Google Scholar (Web of Science—162 citations). These papers could be cited for a number of reasons, but it is very likely that many of the citations that they have received are in effect proxy citations to the NARCCAP data.

This practice of citing a related paper will likely continue to be common for the foreseeable future. In a separate case study, Weber, Mayernik, and Worley [33] found that over 80% of the citations to a series of data release papers for a prominent ocean data set were actually “data use” citations. But as Fig 1 shows, when users of the NARCCAP and NCEP FNL data sets do create references with the persistent identifiers, they are almost entirely referring to the data sets via citations, not acknowledgments or in-text references. This is a notable finding given that acknowledgments and in-text references are common for references that do not include the DOIs. This finding conflicts, however, with the study of Mayo, Vision, and Hull [24], which found that the papers published in association with data sets archived in the Dryad data repository more commonly referenced the Dryad DOI in the text of the paper, not the reference list. (Mayo, Vision, and Hull included the acknowledgment section as part of the body text in their study.)

In the case of software and computing facilities, however, practices for citation are not established to the same extent. While some software packages and computing facilities have accompanying release papers, these are not as common as release papers about data sets. Without a set base of established practices providing inertia, the shift in reference practices from references without identifiers to references with identifiers has occurred much more quickly for the NCL software and Yellowstone supercomputer cases.

Another key takeaway of these cases is that outreach, advocacy, and recommendations to users are also critical for effecting changes in referencing behavior. For example, the NARCCAP data set shows a higher proportion of non-ID references that are citations (right side of Fig 1) than the other three cases. It also shows a bump in total references in 2012. These findings likely correspond to outreach activities on the part of the NARCCAP data manager. In March 2011, the NARCCAP data manager announced an official citation for the NARCCAP data set on the NARCCAP community mailing list (Seth McGinnis, personal communication). This citation announcement was sent out about a year before a DOI was assigned to the data set, which is likely why the number of citations without DOIs is relatively high. In another example, as mentioned above in the methods section, the web page created by the operators of the Yellowstone supercomputer provides recommendations to the community on how to acknowledge their use of the Yellowstone facility via both an acknowledgment and a citation. There is guidance text for both, and the page indicates that users might use the acknowledgment if the citation is “inappropriate.” As shown in Fig 1, the recommended acknowledgment has been used much more than the recommended citation. A hypothesis that emerges from the Yellowstone supercomputer case is that the results would be weighted more towards citations if the associated web page only listed a recommended citation.

Finally, both the NARCCAP data timeline and the NCL software timeline figures show flattening of total reference counts in the last few years. The precise reasons for these trends are unknown, but this discussion raises a few testable hypotheses. It is possible that the flattening of the reference counts for the NARCCAP data set is related to the proxy citations (e.g. citations to data set release papers) and the NARCCAP community outreach noted above. According to the Web of Science citation index, the Mearns et al [32] paper about the NARCCAP data set has increased in citations every year since its publication, having received 37, 41, and 48 citations in the years 2014–2016. This indicates that the overall use of the data set is still increasing, despite what is shown in Fig 2. A hypothesis for future study is that an analysis of the citations to the NARCCAP data set vs. citations to the NARCCAP release papers might show different author groupings. In other words, the initial set of NARCCAP data users (years 2010–2012) may have largely drawn from NARCCAP project participants. As such, they were in regular communication with the NARCCAP data team, and received the requests to reference the data set via citations and the DOI. Later data users may be much more diverse, with more data use coming from individuals outside of the initial project collaboration. As such, they may not receive regular (or any) communications directly from the NARCCAP data management team, and may not be aware of the NARCCAP data citation. These kinds of community factors, however, likely do not apply to the NCL case, which has a relatively long-lasting and widespread user base. The effects of outreach efforts, on the other hand, could be testable, as the NCL development team performs regular tutorials and workshops. Future study could test whether the extent which software citation is included in these training events shows any effect on the subsequent citation rates for the software package.

## Study limitations

Because this study uses a convenience sample and includes a small number of cases, it is more indicative of data citation trends than showing strong validation of any individual trend. We did not attempt to determine statistical significance to the trends due to the small N. Gathering a large number of cases to assess data citation trends is very difficult outside of narrow domains, such as biomedicine (PubMed), physics (arXiv.org), and astronomy (Astronomical Data Service), where most of the literature is available via publicly accessible and machine readable systems. In the geosciences, the relevant domain area for this study, the literature is spread across numerous publisher platforms which do not allow any overarching machine-accessibility capabilities. Google Scholar, while quite comprehensive in coverage, also does not allow any significant automated literature mining. Further study with additional cases and a longer time range is therefore needed to validate the observed trends of this study via statistical evaluation.

One other uncontrollable factor in this study is the potential confounding role of journal editorial policies. Journal policies related to citations and acknowledgments vary widely, and the editors and copy editors for individual journals may enforce the policies in different ways. Journals in some cases ask or require authors to move certain citations to acknowledgments, delete citations, or remove URLs and DOIs. Journals and publishers are a potentially important change agent for data citation practices going forward. Publisher policies and editorial procedures have a direct impact on researchers’ ability to publish their work. As such, publishers, editors, and peer reviewers are in a position to require, recommend, and potentially enforce new behaviors such as data sharing and citation [34]. Some publishers are already moving to make changes on these, including PLOS, which instituted a data availability policy in 2014 (http://journals.plos.org/plosone/s/data-availability). Directly relevant to the cases in this paper, the American Meteorological Society (AMS), which publishes eleven journals in the atmospheric and climate sciences, added a “Data Archiving and Citation Recommendation” to its author guidelines in early 2015 (http://www.ametsoc.org/PubsDataPolicy, see also [7]). The recommendation details potential places to archive data, and provides information on how to create a data citation, including an extensive list of examples. The AMS recommendation would not have impacted the study presented in this paper because of the lag time for journal publications. Papers published in AMS journals in the calendar year 2015 would have been submitted, revised, and accepted before the recommendation was added to the author guidelines. Future study will be necessary to determine the impacts that such a policy might have on the data citation behaviors of its target communities.

## Conclusion

This paper uses four focused case studies to evaluate whether changes in the practices of researchers can be detected with regard to the citation of research infrastructural resources: data sets, software packages, and research facilities. In particular, this investigation examined the extent to which research resources are being increasingly identified and referenced in the research literature via persistent citable identifiers like DOIs and ARKs. The findings of the four case studies show that the use of persistent identifiers are increasing in references to research infrastructural resources, but that the patterns of these increases are variable across the four cases. This study suggests that citation practices for data sets may change more slowly than citation practices for software and research facilities, due to the inertia of existing practices for referencing the use of data, such as by citing a published article. Similarly, the existing practice of acknowledging computing support may slow the adoption of formal citations for computing resources.

The manual search process undertaken in this study was very effective but limited in scope. When conducting this kind of study through the use of general web search engines like Google, papers that do reference data or software with the persistent identifiers are much easier to find than papers that reference such resources by name or in an indirect fashion. This is, of course, one of the obvious benefits of assigning such persistent IDs. But as illustrated by the findings in this paper, there will continue to be many papers published that do not reference research resources by their assigned persistent IDs. The chronic problems of undercounting related to these inconsistent referencing practices will reduce the potential utility of such citation metrics for the managers of these resources, policy makers who fund them, or bibliometric researchers trying to study data citation behavior over time.

The overall trends related to the citation of research infrastructures, however, appear to be positive. Continued education and outreach efforts on the parts of data centers, research funders, and information professionals will hopefully accelerate the change rates of these trends.
